# Cardiovascular magnetic resonance imaging characteristics of a myocardial metastatic melanoma

**DOI:** 10.1093/ehjci/jead326

**Published:** 2023-11-22

**Authors:** Luuk H G A Hopman, Irene M Frenaij, Josephine F Heidendael, Jasper L Selder, Lourens F H J Robbers

**Affiliations:** Department of Cardiology, Amsterdam UMC, De Boelelaan 1118, 1081 HV Amsterdam, The Netherlands; Department of Cardiology, Amsterdam UMC, De Boelelaan 1118, 1081 HV Amsterdam, The Netherlands; Department of Cardiology, Amsterdam UMC, De Boelelaan 1118, 1081 HV Amsterdam, The Netherlands; Department of Cardiology, Amsterdam UMC, De Boelelaan 1118, 1081 HV Amsterdam, The Netherlands; Department of Cardiology, Amsterdam UMC, De Boelelaan 1118, 1081 HV Amsterdam, The Netherlands

A patient with metastatic melanoma showed disease progression during third-line immunotherapy. To further assess the situation, a positron emission tomography-computed tomography (PET-CT) scan was conducted, revealing extensive metastatic disease involving the liver, adrenal glands, skeleton, and lymph nodes. Notably, a conspicuously fluorodeoxyglucose-avid paraseptal lesion emerged in the right ventricle (RV), prompting concerns about potential cardiac metastasis of the melanoma (*Panels A* and *B*). Despite probable disease progression affecting the heart, the patient, who has no documented history of cardiac disease, showed no clinical signs of heart failure or arrhythmia. Subsequent echocardiography identified a paraseptal RV mobile mass but could not differentiate between thrombus and tumour (*Panels C* and *D*). Consequently, a cardiac magnetic resonance (CMR) scan was performed for further characterization.

**Figure:**
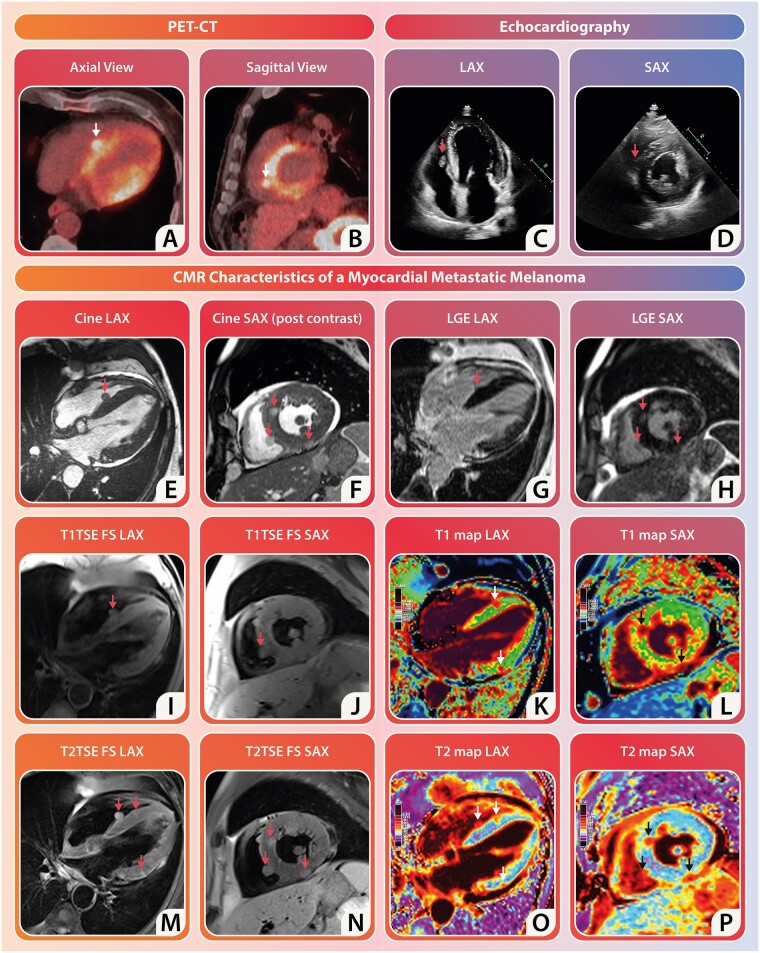


Cardiac magnetic resonance cine-imaging revealed a left ventricular (LV) function of 39%, indicating global hypokinesia and heterogeneous thickening of the LV myocardial wall (please see Supplementary data online, *Material* for cine movies). Conversely, the RV dimensions and function appeared normal with an ejection fraction of 53%. Prominent pericardial effusion was observed, along with a spherical lesion previously detected via echocardiography at the mid-ventricular RV-septum (*Panel E*). Short-axis cine-images after contrast administration showed multiple spherical lesions, approximately measuring 11 × 11 mm, in the inferior and anterior LV regions, some protruding into the pericardial space (*Panel F*). Tissue characterization indicated that these lesions were isointense to the myocardium on T1-weighted images (*Panels I* and *J*). Cardiac metastases typically present as hypointense on T1-weighted images, yet melanoma often deviates from this norm due to the presence of paramagnetic metals bound by melanin, explaining the isointensity of the lesions rather than the expected hypointensity in other malignancies. The lesions were hyperintense on T2-weighted images (*Panels M* and *N*), possibly indicating oedema or neovascularization. While T1 mapping exhibited a modest increase in values across the heart (1085 ± 75 ms), the lesions themselves exhibited significantly elevated T1 values (1393 ± 93 ms) (*Panels K* and *L*). T2 mapping produced normal values (48.2 ± 6.3 ms), with the lesions showing increased T2 values (73.6 ± 7.9 ms) (*Panels O* and *P*). Late gadolinium enhancement (LGE) images showed non-ischemic contrast distribution with LGE in the intramyocardial round lesions (*Panels G* and *H*), which may result from extracellular matrix architecture changes often seen in the melanoma tumour microenvironment.

Concluding, in the diagnosis of cardiac metastatic melanoma, CMR proves to be a valuable addition to PET-CT and echocardiography, enhancing the ability to detect active spherical lesions within the myocardium.

## Supplementary Material

jead326_Supplementary_Data

